# Identification of novel antigen candidates for a tuberculosis vaccine in the adult zebrafish (*Danio rerio*)

**DOI:** 10.1371/journal.pone.0181942

**Published:** 2017-07-25

**Authors:** Henna Myllymäki, Mirja Niskanen, Kaisa Ester Oksanen, Eleanor Sherwood, Maarit Ahava, Mataleena Parikka, Mika Rämet

**Affiliations:** 1 BioMediTech Institute and Faculty of Medicine and Life Sciences, University of Tampere, Tampere, Finland; 2 Oral and Maxillofacial Unit, Tampere University Hospital, Tampere, Finland; 3 PEDEGO Research Unit, Medical Research Center Oulu, University of Oulu, Oulu, Finland, and Department of Children and Adolescents, Oulu University Hospital, Oulu, Finland; Fundació Institut d’Investigació en Ciències de la Salut Germans Trias i Pujol, Universitat Autònoma de Barcelona, SPAIN

## Abstract

Tuberculosis (TB) remains a major global health challenge and the development of a better vaccine takes center stage in fighting the disease. For this purpose, animal models that are capable of replicating the course of the disease and are suitable for the early-stage screening of vaccine candidates are needed. A *Mycobacterium marinum* infection in adult zebrafish resembles human TB. Here, we present a pre-clinical screen for a DNA-based tuberculosis vaccine in the adult zebrafish using an *M*. *marinum* infection model. We tested 15 antigens representing different types of mycobacterial proteins, including the Resuscitation Promoting factors (Rpf), PE/PPE protein family members, other membrane proteins and metabolic enzymes. The antigens were expressed as GFP fusion proteins, facilitating the validation of their expression *in vivo*. The efficiency of the antigens was tested against a low-dose intraperitoneal *M*. *marinum* infection (≈ 40 colony forming units), which mimics a primary *M*. *tuberculosis* infection. While none of the antigens was able to completely prevent a mycobacterial infection, four of them, namely RpfE, PE5_1, PE31 and cdh, led to significantly reduced bacterial burdens at four weeks post infection. Immunization with RpfE also improved the survival of the fish against a high-dose intraperitoneal injection with *M*. *marinum* (≈ 10.000 colony forming units), resembling the disseminated form of the disease. This study shows that the *M*. *marinum* infection model in adult zebrafish is suitable for the pre-clinical screening of tuberculosis vaccines and presents RpfE as a potential antigen candidate for further studies.

## Introduction

Tuberculosis (TB) remains a major health problem that has been extensively studied in recent years. *Mycobacterium tuberculosis*, the causative agent of TB, caused 1.4 million deaths and 10.4 million new infections in 2015 [1]. The prevalence of TB is highest in Africa and Asia, where 75% of all new cases are diagnosed [1]. The World Health Organization (WHO) estimates that one third of the human population have a latent TB infection and carry up to a 10% lifetime risk of reactivation into an active disease [1]. In addition, the multi-drug resistant *M*. *tuberculosis* strains and HIV co-infections hamper the treatment of TB [1,2].The WHO has set an ambitious goal to eliminate TB as a global health problem by the year 2050 [1]. To reach the goal, new innovative approaches are needed.

Interest in developing novel tuberculosis vaccines has grown over the years. The only available TB vaccine, Bacillus Calmette Guérin (BCG), protects young children, but its ability to induce long-term cell mediated immune responses varies and the protection it provides against pulmonary TB or against the reactivation of latent TB is limited [3–5]. Therefore, new vaccines that protect from the primary infection, boost BCG induced immunity or prevent the reactivation of a latent infection, are needed to overcome TB.

A central issue in TB research has been the paucity of good animal models [6]. *M*. *tuberculosis* is not a natural pathogen of traditional animal models such as mice, rabbits and guinea pigs, and natural hosts, non-human primates, can be used only very selectively for experiments [6]. In the past ten years, the zebrafish (*Danio rerio*) has emerged as an advantageous animal to model a TB infection. An infection with *Mycobacterium marinum*—a close relative to *M*. *tuberculosis*—in zebrafish leads to a disease that resembles human TB in many aspects [7]. *M*. *marinum* is a natural pathogen of fish and an infection can lead to either an active or a naturally latent form of the disease [8–10]; reviewed in [11]. As a vertebrate, the zebrafish has both an innate and an adaptive immunity with essentially the same immune cell populations as are present in humans, including neutrophils, macrophages and both T and B cells. Also, zebrafish CD4+ and CD8+ lymphocytes perform similar functions as in humans [12–17]. Although there are physiological differences between humans and zebrafish, most importantly fish lack lungs and are smaller than humans, there is accumulating evidence for the similarities in immune responses involved in mycobacterial infections in zebrafish and humans, and factors increasing susceptibility to infections [17–26]. In addition, similar virulence factors and immune evasion strategies are used by both *M*. *marinum* and *M*. *tuberculosis* [19,27–31]. The data obtained from the zebrafish studies has already proven useful in the design of novel drugs and therapies against TB [21,25,30,32].

Despite the increasing knowledge on mycobacterial pathogenesis, the development of new TB vaccines has turned out to be challenging. Currently there are 14 vaccine candidates in the clinical trial pipeline, including inactivated or attenuated whole-cell vaccines, and subunit vaccines containing mycobacterial antigens. [4,33]. Expression of a bacterial antigen leads to the production of cytokines, including Interferon gamma (IFN-γ), and antigen presentation via the major histocompatibility complex of dendritic cells and the development of antigen specific memory cells [34]. An important advantage of DNA vaccines over BCG and other live attenuated vaccines is that they can be safely administered to immunocompromised people. [1,4,35].

A key step in the design of DNA vaccines is the choice of the antigen(s), especially since DNA vaccines tend to have a relatively weak immunogenicity in humans [34]. Even though there are methods for predicting the immunogenicity of selected antigens, *in vivo* infection models are required to assess the efficacy of the novel vaccine candidates as there are currently no reliable biomarkers for predicting the efficacy of protection against TB [4,36,37]. We have previously shown that adult zebrafish can be partially protected against mycobacteriosis with the BCG vaccine or with a DNA vaccine expressing a combination of antigens [38,39]. The current study was designed to test the applicability of the adult zebrafish-*M*. *marinum* infection model in the pre-clinical screening of DNA-based tuberculosis vaccines. Based on literature and online databases, MarinoList and TubercuList [40,41], we selected 15 *M*. *marinum* antigens that have a homologue in *M*. *tuberculosis* and predicted or experimentally shown immunogenicity. We selected molecules that belong to different functional categories and are expressed during different stages of mycobacterial growth, including four Resuscitation promoting factors [42], three PE/PPE family members [43] and five other membrane associated proteins together with three proteins involved in metabolism. The selected antigens were tested as prophylactic DNA vaccines using two variations of the zebrafish mycobacterium infection model: a low-dose infection that mimics a primary TB infection leading to latency; and a high-dose infection that replicates miliary tuberculosis.

## Materials and methods

### Fish

Adult (5–7 month-old) wild type AB zebrafish were used for all experiments and maintained as in (Parikka et al, 2012). Animal studies were approved by the National Animal Experiment Board in Finland (Approval number ESAVI/8125/04.10.07/2013) and conducted in accordance with the EU-directive 2010/63/EU on the protection of animals used for scientific purposes.

### Culture of *M*. *marinum* and qRT-PCR

The *Mycobacterium marinum* strain ATCC 927 was cultured on 7H10 Middlebrook OACD plates (BD Biosciences, Franklin Lakes, NJ) at +29°C, inoculated to a fresh plate every 7 days, and a fresh stock was thawed after every two passages. Liquid cultures for RNA isolation and infections were grown in 7H9 Middlebrook medium (BD Biosciences, Franklin Lakes, NJ), see below for details.

Expression of the *M*. *marinum* genes corresponding to the selected antigens was confirmed by qRT-PCR from *M*. *marinum* RNA. The *M*. *marinum* ATCC 927 strain was cultured in 7H9 (BD Biosciences) medium to the log phase (OD600 of 0.6). Bacteria from six separate liquid cultures were collected by centrifuging for 5 minutes at 800 x g. The pellets were used for RNA extractions with the RNeasy Mini Kit (Qiagen) according to the manufacturer’s instructions. Before qRT-PCR, the RNA samples were treated with DNase (RapidOut DNase Removal kit, Thermo Fischer Scientific, Waltham, MA USA). The expression of mycobacterial genes was verified with the iScript™ One-Step RT-PCR Kit with SYBR® Green (Bio-Rad, California, USA) according to the manufacturer’s instructions. The *M*. *marinum internal transcribed spacer* (*MMITS*) [8] was used as a reference gene, and the qRT-PCR results were analyzed by the ΔCt-method [44]. The primers used for qRT-PCR were designed using the Primer3Plus software [45] and are listed in S1 Table.

### Construction of DNA vaccines and immunizations

Homology between the *M*. *tuberculosis* and *M*. *marinum* genes was analyzed with the Clustal Omega sequence alignment tool [46]. The cellular location of the chosen *M*. *marinum* proteins was determined based on experimental evidence available in the literature or by prediction of transmembrane protein topology with a hidden Markov model [47]. Antigen sequences of different lengths were selected for expression in the candidate vaccine, however, when possible, all or part of the extracellular region of the *M*. *marinum* protein was included in the vaccine antigen. The Expasy Compute pI/Mw tool [48] was used to calculate the expected molecular weight of the antigen-GFP fusion proteins. The primers used for cloning the antigens are listed in S2 Table.

DNA vaccine constructs were prepared and the DNA vaccinations performed as described in [38]. In brief, the chosen antigen regions were amplified from *Mycobacterium marinum* ATCC grown on 7H10 Middlebrook OACD plates (BD Biosciences, Franklin Lakes, NJ) by colony PCR. Purified PCR products were restriction cloned into the pCMV-*eGFP* expression vector to be expressed with a C-terminal GFP tag (Addgene plasmid 11153), transformed into *E*. *coli* One Shot TOP10 cells (Invitrogen) and confirmed by sequencing. For DNA immunizations, plasmid DNA was purified using the QIAGEN Plasmid Plus Maxi Kit (Qiagen, Venlo, The Netherlands). The pCMV-EGFP plasmid without mycobacterial inserts was used for control vaccinations.

For vaccine immunizations, the fish were briefly anaesthetized in 0.02% 3-aminobenzoic acid ethyl ester (pH 7.0) (Sigma–Aldrich) and injected in the dorsal muscle with 12 μg of the vaccine or the *pCMV*-eGFP plasmid using aluminosilicate capillary needles and a PV830 Pneumatic PicoPump microinjector (World Precision Instruments, Sarasota, FL). The injection was followed by electroporation (6 pulses, 40 V, 50 m s each) using the GenePulser-electroporator (Bio-Rad, Hercules, CA) with tweezer-type electrodes (BTX/Harvard Apparatus, Holliston, MA) [39].

### Fluorescence microscopy, Western blotting and GFP ELISA

*In vivo* expression of the plasmid DNA-derived protein products (GFP and its antigen recombinants) was verified by fluorescence microscopy, Western blotting and ELISA using naïve fish as a negative control. Nikon AZ100 fluorescent microscope was used for the microscopy. For Western blotting and ELISA, the fish were dissected under UV light and their dorsal muscles that showed the fluorescence indicative of vaccine antigen expression were collected for analysis. The samples were homogenized in TriReagent (Molecular Research Centre, Inc., Cincinnati OH, USA) with ceramic beads (MO BIO Laboratories, Carlsbad CA, USA) using a PowerLyzer™ 24 Bench Top Bead-Based Homogenizer (MO BIO Laboratories), followed by a protein extraction protocol according to the manufacturer’s instructions.

The Pierce^®^ BCA Protein Assay Kit (Thermo Fisher Scientific, Waltham, MA) was used to define the total protein concentration of each lysate. For Western blotting, a volume corresponding to a total protein amount of 7.5–15 **μ**g of each fish homogenate was resolved on a 4–20% Mini-PROTEAN^®^ TGX™ Gel (BioRad) and blotted onto a nitrocellulose membrane using Trans-Blot^®^ Turbo™ Mini Nitrocellulose Transfer Packs (BioRad). The horse radish peroxidase conjugated GFP Tag Monoclonal Antibody (GF28R) (Thermo Fisher) was used for detection of the target protein. The GFP ELISA Kit (Cell Biolabs, San Diego, CA) was used for determining the relative levels of GFP according to the manufacturer’s instructions. The absorbance values were transformed into GFP concentrations using a GFP standard, and the amount of GFP in each sample was normalized with the total protein concentration of the sample and with the average of the GFP controls in the experiment. Non-immunized AB fish were used as a negative control in both Western blots and ELISA.

### *M*. *marinum* infections

Fish were infected either with a low (~40 cfu) or high (~10,000 cfu) dose of *M*. *marinum* four weeks after immunization. *M*. *marinum* ATCC 927 was cultured at 29°C in standard mycobacterium medium, 7H9 (BD Biosciences), and prepared for infections as described in [8]. For infections, fish were anesthetized with 0.02% 3-aminobenzoic acid ethyl ester. The desired dose of *M*. *marinum* diluted in 0.2 M sterile KCl was injected intraperitoneally (i.p.). Thereafter, the fish were immediately released into a recovery tank. Infection doses were verified by plating the bacteria onto 7H10 plates (BD Biosciences). Following the infections, the well-being of the fish was monitored daily, and fish showing signs of stress or mycobacterial disease during the experiment follow-up period were euthanized with 0.04% 3-aminobenzoic acid ethyl ester.

### Nucleic acid extraction and quantification of bacterial burdens

To assess vaccine efficacy on a primary infection, AB fish immunized with experimental or control (GFP) antigens (~15 fish/group) were infected with a low dose (~40 cfu) of *M*. *marinum* four weeks post-immunization. Five weeks after infection, the fish were subjected to DNA extraction. Fish showing signs of disease during the five-week follow-up interval were euthanized immediately and included in the cfu analysis. The contents of the peritoneal cavity, including the visceral organs, of euthanized fish was collected into homogenization tubes (Mobio, California, USA) and homogenized in 1.5 ml of TRI reagent (MRC, OH, USA) using the PowerLyzer24 bead beater (Mobio). Homogenized samples were sonicated using an m08 water bath sonicator (Finnsonic, Lahti, Finland) and DNA extractions were then carried out as in [38]. The bacterial burden per fish was measured from the DNA samples by qPCR with *M*. *marinum*-specific primers using a standard curve with previously determined bacterial loads as described in [8]. Antigen immunizations that showed a protective effect (or tendency) were repeated one or two more times with similarly sized groups.

### Survival follow-up

For survival experiments, the control and experimental fish (19–34 fish/group) were infected with a high dose (~10.000 cfu) of *M*. *marinum* and followed for twelve weeks. Fish included in the survival experiments were monitored daily for their well-being and humane end point criteria ratified by the national ethical board were followed. Fish showing signs of discomfort or disease were euthanized using 0.04% 3-aminobenzoic acid ethyl ester. Antigen immunizations that showed a protective effect (or tendency) (RpfE, PE31, MMAR_3501, esxM, cdh) were repeated one or two more times with similarly sized groups.

### Power calculations and statistical analyses

The required sample size (n) for each experiment was calculated using the following formula: $n={2(Z_{a}+Z_{1-\beta })}^{{2\sigma }^{2},}/\Delta^{2}$, in which Z_α_ (1.96) is a constant set based on the accepted error α (0.05), Z_1-_β (0.8416) is a constant set according to the power of the study (0.8), σ is the estimated standard deviation (0.5). Δ is the difference in the effects of the two treatments compared (estimated effect size), and was set to 0.5 (50%) relating to a reduction in the bacterial burden or improvement in the survival percentage. This is approximately the same as the effect that is achieved by the BCG vaccination [38,39]. Based on these calculations, the minimum group size was set to 14 fish [49].

Statistical analyses were done using the GraphPad Prism 5.02 software (GraphPad Software Inc., California, USA). The statistical tests used were the log rank Mantel–Cox test for the survival experiments, and the Mann–Whitney test for bacterial counts and ELISA results. Values of p≤0.05 were considered significant.

## Results

### Choice of antigens and antigen construction

For the vaccine antigen screen, we selected genes that belong to diverse functional categories and are expressed at different stages of the mycobacterial life cycle. In addition, we chose antigens with different (observed or predicted) cellular locations, although we focused on secreted and membrane-associated proteins, as these presumably are more likely to elicit responses by the host immune system [50]. We chose the antigens based on literature (see below) and homology data in online databases Tuberculist [40] and Marinolist [41].

Resuscitation promoting factors (Rpf) are proteins with peptidoglycan-hydrolysing activity and are thought to be important for mycobacterial virulence and especially for resuscitation from dormancy. Mutant bacterial strains with *Rpf* deficiencies display defects in replication, reactivation and in persistence to stress, presumably due to alterations in the structure of their cell wall [10,51–53]. There are five *rpf* genes in the *M*. *tuberculosis* genome (*rpfA-E*) [42] and four in *M*. *marinum* (*rpfA*, *-B* and -*E*, and *resuscitation-promoting factor-like protein* (*mmar_2772*) homologous to *M*. *tuberculosis rpfC*, which is hereafter referred to as *rpfC*). Despite the name, the expression profiles of the *M*. *tuberculosis rpf* genes differ according to the infection phase, suggesting that they have distinct functions [54]. As Rpfs have also been reported to have immunogenic properties in mice [55] and in humans [56]; for a review see [57], we included all four *M*. *marinum* antigens in our screen.

Another relatively well-studied group of potential mycobacterial antigens are the PE/PPE proteins, which are named after the proline-glutamic acid (PE) and proline-proline-glutamic acid (PPE) motifs near their N-termini. The *pe*/*ppe* genes constitute ∼10% of the genome of pathogenic mycobacteria, and their expression is differentially regulated by stress and other environmental conditions, including inside granulomas. PE/PPE proteins are commonly localized to the bacterial cell surface or are secreted, enabling them to elicit and modulate host immune responses. Many PE/PPE proteins have been shown to be highly antigenic [58] and several have been studied as vaccine candidates, of which a candidate comprising a polyprotein of Mtb32 (PepA) and Mtb39 (PPE18) has progressed to clinical studies [59]. For our screen, we chose three members of this protein family that had not yet been tested as vaccine candidates, namely PE5_1, PE19_1 and PE31.

We also included proteins with a signature expression profile in certain phases of the infection. For this, we chose the outer membrane protein A (ompA), whose homolog in *M*. *tuberculosis* induces strong IFN-γ responses in cattle [60], and the predicted transmembrane protein MMAR_3501 encoded by the Dormancy survival regulon (DosR), that is highly immunogenic, especially in patients with LTBI [61]. In addition, we selected the Early secreted antigenic target (ESAT)-6/10-kDa culture filtrate protein (CFP-10) family member esxM, whose homolog in *M*. *tuberculosis*, Rv3620c, is a secreted, antigenic protein [62]; the lipoprotein lprG, whose homolog in *M*. *tuberculosis* has been shown to induce the activation of memory T cells in humans [63]; and MMAR_4207, a predicted transmembrane protein of unknown function with a highly conserved homolog in *M*. *tuberculosis* [40,41].

Knowing that mycobacteria undergo extensive metabolic changes during the different stages of their lifecycle [64], we chose components of metabolic pathways as vaccine antigens. The biosynthesis of cysteine is needed in the oxidative defense and for dormant mycobacteria to persist inside infected macrophages. Therefore, we selected cysQ and cysM, two critical enzymes of this pathway [65,66]. In addition, we chose cdh, a predicted membrane protein and CDP-diacylglycerol pyrophosphatase, which is involved in the biosynthesis of phospholipids, and whose *M*. *tuberculosis* homolog Rv2289 shows high abundance in the virulent H37Rv strain, but is nearly absent from the avirulent H37Ra strain [67].

The selected antigens together with their *M*. *tuberculosis* homologs are listed in Table 1. For clarity, the same grouping according to the (predicted) function of the antigen proteins will be used throughout the paper.

**Table 1:** The selected antigens and their *M*. *tuberculosis* homologs with predicted functions.

Accessionnumber	Protein name	*M*.tuberculosis protein (% homology)	Protein Size (aa)	Predicted protein function	Reference
**Resuscitation Promoting Factors**					
MMAR_4665	RpfA	Rv0867c (84%)	386	Peptidoclycan hydrolase. May promote the resuscitation of dormant cells.	[42,51,53]
MMAR_4479	RpfB	Rv1009 (85%)	363	Peptidoclycan hydrolase. May promote the resuscitation of dormant cells.	[8,42,52]
MMAR_2772	resuscitation-promoting factor-like protein	Rv1884c/RpfC (66%)	138	Peptidoclycan hydrolase. May promote the resuscitation of dormant cells.	[54–56]
MMAR_3776	RpfE	Rv2450c (74%)	244	Peptidoclycan hydrolase. May promote the resuscitation of dormant cells.	[40,41,57]
**PE/PPE proteins**					
MMAR_5258	PE5_1	Rv1386/PE15 (70%)	103	Membrane protein of unknown function.	[43,58]
MMAR_2670	PE19_1	Rv1788/PE18 (89%)	99	Membrane protein of unknown function.	[43,58]
MMAR_4241	PE31	Rv1195/PE13 (70%)	99	Membrane protein of unknown function.	[43]
**Transmembrane proteins and secreted factors**					
MMAR_4207		Rv1234 (95%)	175	Conserved hypothetical membrane protein of unknown function.	[40,41]
MMAR_3501		Rv1733c (38%)	193	Conserved hypothetical membrane protein of unknown function.	[61]
MMAR_4637	ompA	Rv0899/ompA (67%)	332	Structural outer membrane protein that may protect the integrity of the bacterium.	[60]
MMAR_2674	esxM	Rv3620c/esxW (87%)	98	Secreted, ESAT-6/CFP-10 family protein, function unknown.	[62]
MMAR_2220	lprG	Rv1411c/lprG (78%)	233	Conserved lipoprotein of unknown function.	[63]
**Metabolic enzymes**					
MMAR_3112	cysQ	Rv2131/cysQ (78%)	263	Monophosphatase involved in sulphur metabolism.	[65,66]
MMAR_4629	cysM	Rv1336/cysM (76%)	314	Cysteine synthase.	[65,66]
MMAR_3445	cdh	Rv2289 (68%)	264	Secreted CDP-diacylglyserol pyrophosphatase involved in phospholipid biosynthesis.	[67]

### Expression of selected antigens in *M*. *marinum*

**Fig 1:**
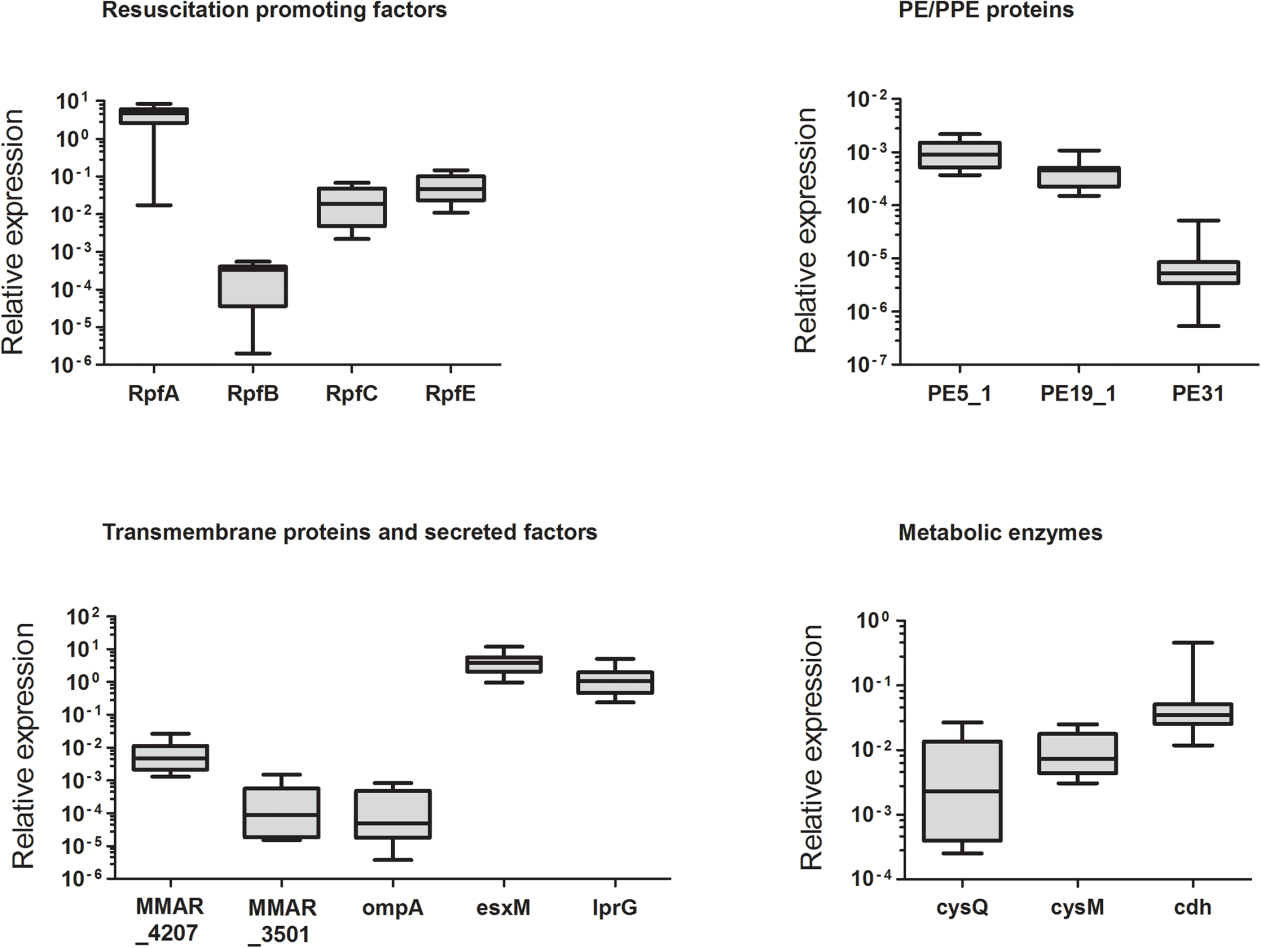
Expression of the antigens in the *M*. *marinum* ATCC 927 strain. A liquid culture of *M*. *marinum* was grown to a log phase, bacteria were harvested by centrifugation and subjected to RNA extraction and DNase treatment. Antigen expression was confirmed by qRT-PCR using primers specific for each antigen (S1 Table). The *M*. *marinum* transcribed internal spacer (*MMITS*) was used as a reference gene. The horizontal lines represent medians and the bars and whiskers represent minimum and maximum values. N = 6.

The expression of the selected *M*. *marinum* genes in the ATCC 927 strain was verified by qRT-PCR (Fig 1). For this purpose, we used a log phase bacterial culture (OD600 of ~0.6), which represents actively growing, infectious mycobacteria. Although the relative expression levels of the candidate genes observed in our bacterial culture varied in a range of a ten times higher expression (*RpfA* and *esxM*) to 10^−5^ (*pe31*) compared to the reference gene *MMITS* expression, each of the selected genes was verified to be expressed. Based on this, all 15 antigens were selected for further studies and cloned into an expression vector as GFP–tagged fusion proteins.

### Verification of antigen expression by the vaccines

**Fig 2:**
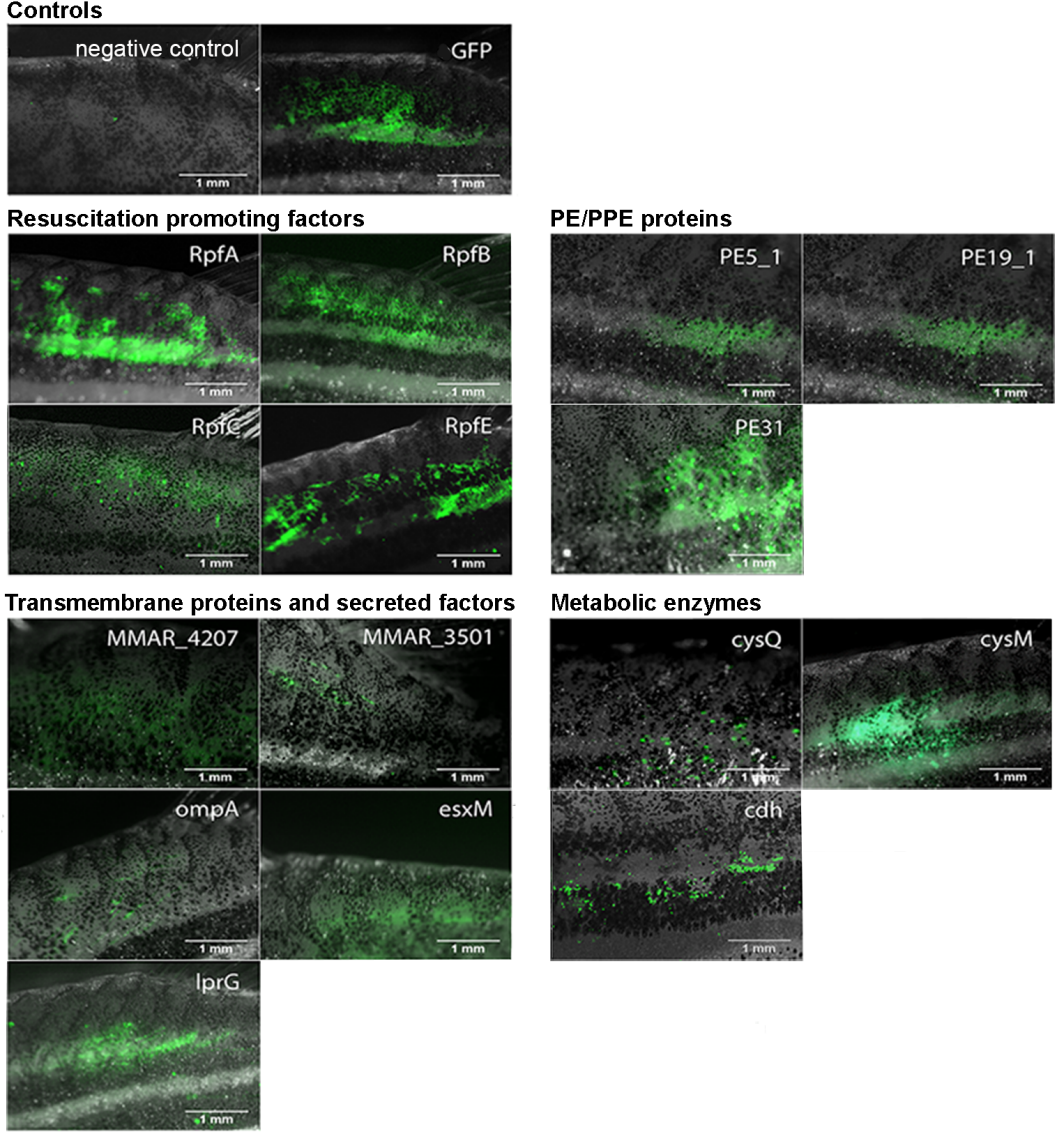
In situ GFP expression in immunized zebrafish. AB fish were immunized with 12 μg of experimental or control vaccine plasmids, followed by electroporation. Seven days post-injection, the successful vaccinations and expression of the antigen-GFP fusion proteins were verified by fluorescence microscopy. The fluorescence resulting from the expression of the antigen-GFP fusion protein is seen in the dorsal muscle near the injection site. For each antigen, a representative example is shown. Non-immunized AB fish were used as a negative control.

One of the key issues in DNA vaccination is achieving adequate antigen expression in the target tissue. To assess this, the in vivo expression of the vaccine constructs was analyzed with three different methods, each utilizing the GFP tag fused with the antigen. First, antigen expression was visualized in situ, in the dorsal muscles of the fish, with a fluorescent microscope. Although not quantitative, visual inspection provides a quick and easy way to evaluate successful vaccinations and antigen expression in each individual fish without harming them. As shown in Fig 2, all of the tested antigens showed detectable GFP expression seven days after vaccination. Fluorescence above the background level of non-immunized fish was observed for all of the candidate antigens, and this was always located near the injection site in the dorsal muscle.

**Fig 3:**
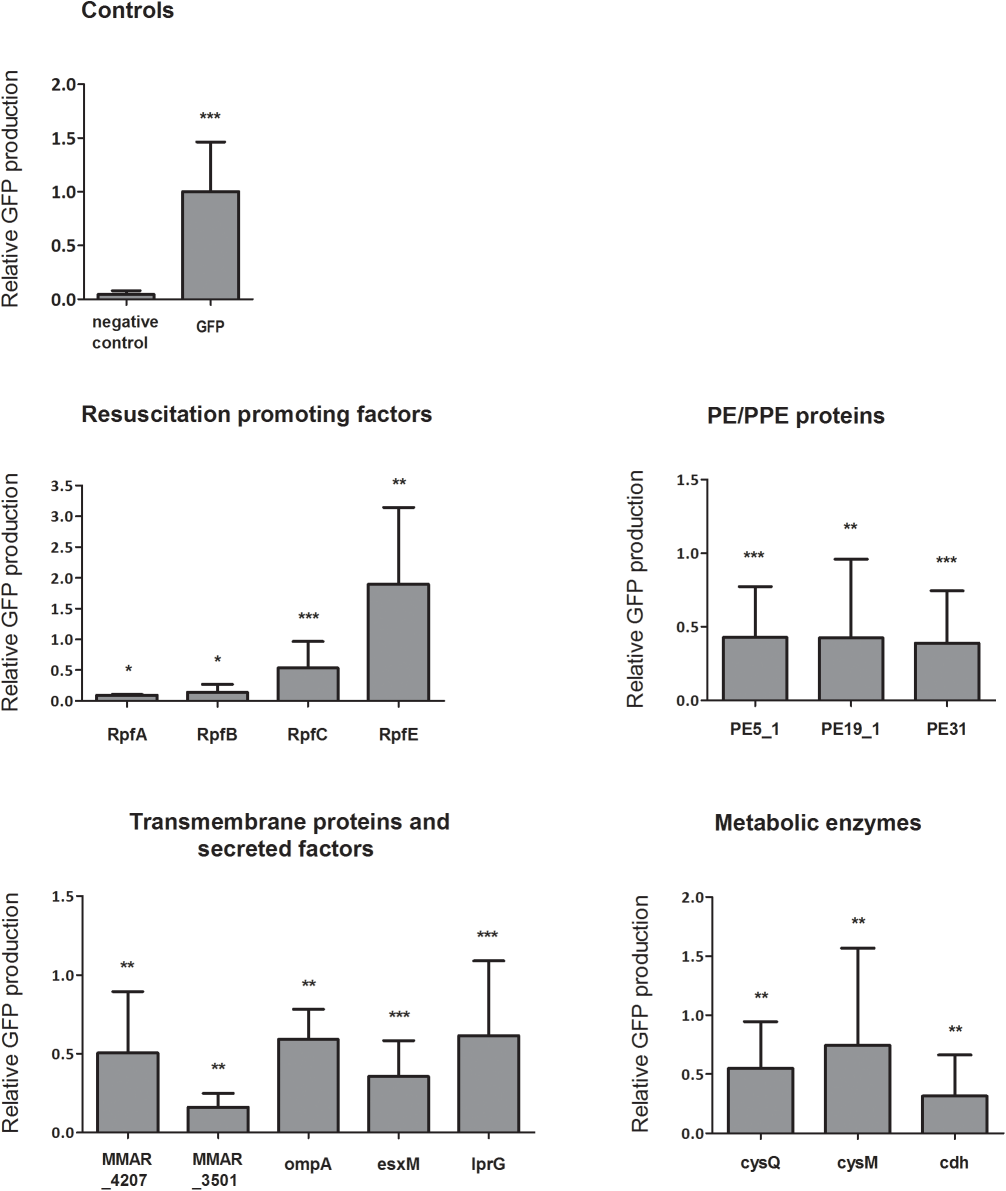
Quantification of mycobacterial antigen expression with GFP ELISA. AB fish were immunized with 12 μg of experimental or control vaccine plasmids, followed by electroporation. Seven days post-injection, fish were dissected under a UV light and the dorsal muscles were collected and homogenized with ceramic beads, followed by protein extraction. 7.5–15 μg of each protein lysate in a 1% SDS buffer was used for a GFP ELISA analysis. A standard curve was used to quantify the absorbance values, which were then normalized with the average of the control values of each experiment before the values were pooled. Non-immunized AB fish were used as the negative control. Mean±SD is shown. N≥4 per group. * p<0.05, ** p<0.01, *** p<0.001 (Two-tailed Mann-Whitney test).

To quantify antigen expression, a GFP enzyme-linked immunosorbent assay (ELISA) was used for proteins extracted from the dorsal muscles of the vaccinated fish. The quantitated expression of the recombinant constructs relative to the GFP control (samples from fish injected with an empty plasmid) is shown in Fig 3. All immunizations led to quantifiable GFP expression. Most antigens had expression levels comparable to the GFP control, while RpfA, RpfB and MMAR_3501 antigens had a rather low expression (10–16% of the GFP control), and the RpfE fusion protein showed expression levels exceeding that of the GFP control.

**Fig 4:**
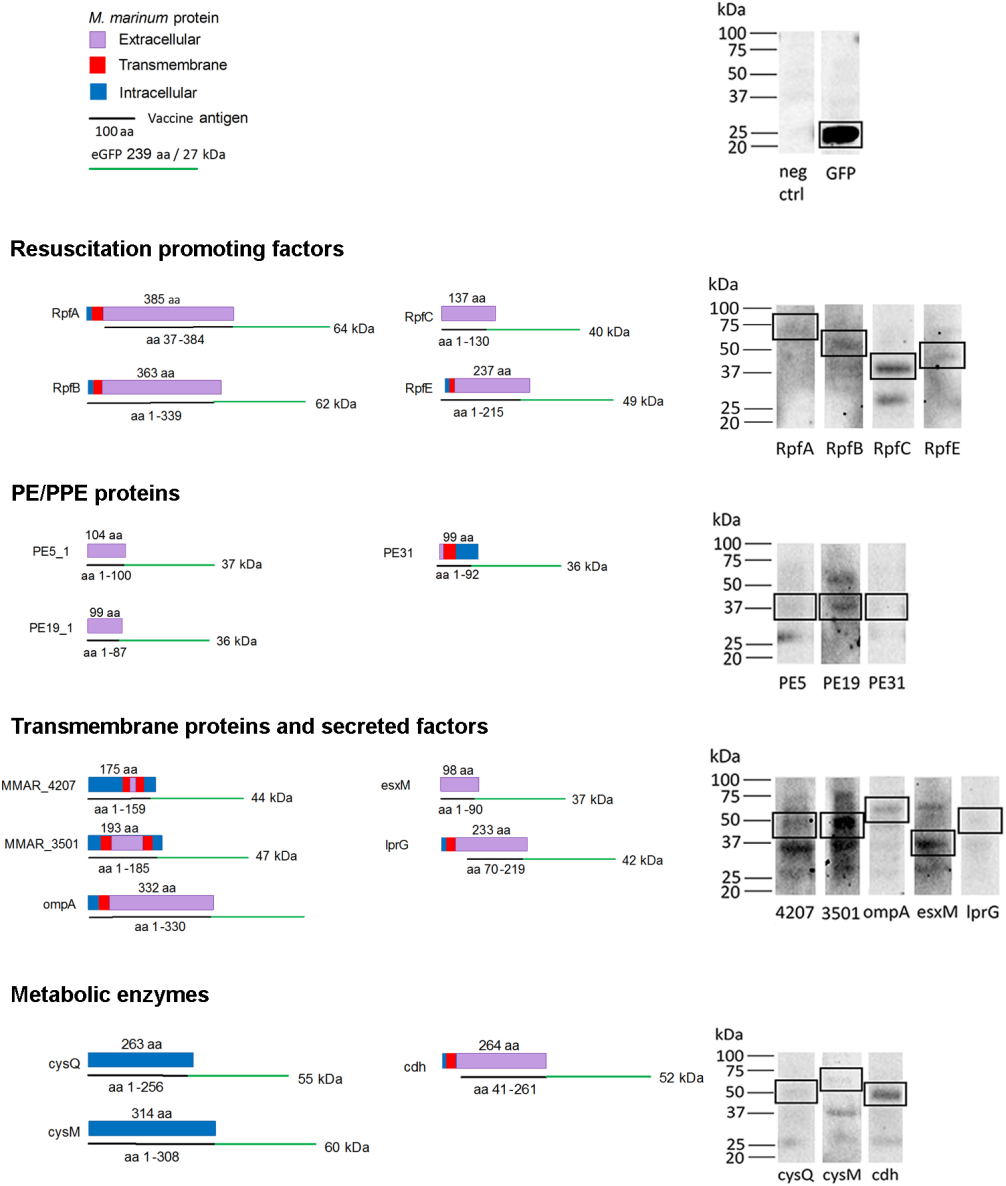
Schematic representation of the vaccine antigens. The *M*. *marinum* proteins are represented by bars, different colors indicate cellular location based on the literature and/or Trans Membrane prediction using Hidden Markov Models (TMHMM). The vaccine antigen-GFP fusion proteins are represented by lines, together with their expected molecular weights (See legend for more details). On the right, an immunoblot analysis of antigen-GFP fusion proteins. For the analysis, AB fish were immunized with 12 μg of experimental or control (empty plasmid with GFP only) vaccines, followed by electroporation. Seven days post-injection, fish were dissected under UV light and the dorsal muscles were collected and homogenized, followed by protein extraction. 7.5–15 μg of each protein lysate was run on an SDS-PAGE gel, blotted onto a nitrocellulose membrane followed by immunodetection with a horse radish peroxidase (HRP) conjugated anti-GFP antibody. Non-immunized AB fish were used as the negative control.

To validate the correct size of the recombinant antigen fusion proteins, they were visualized with Western blotting, using a HRP-conjugated GFP antibody (Fig 4). The GFP control (fish immunized with an empty plasmid) produced a strong band of the expected size (GFP protein, 27 kDa). Importantly, expression of all of the antigens resulted in a detectable band corresponding to the calculated molecular weight of the GFP fusion protein (Fig 4).

### Vaccine efficiency against a low-dose *M*. *marinum* infection

**Fig 5:**
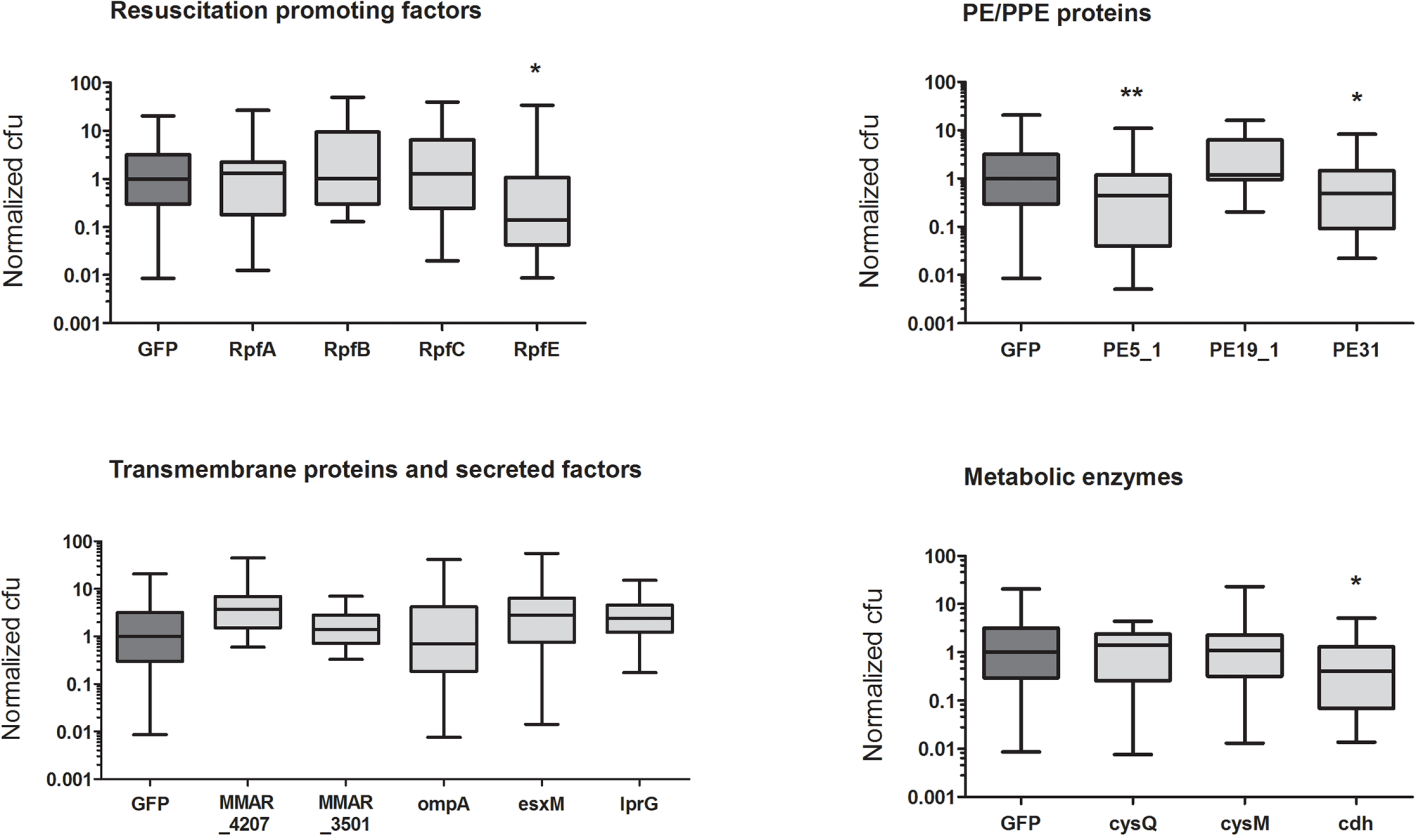
RpfE, PE5_1, PE31 and cdh antigens reduce bacterial burdens in adult zebrafish infected with a low-dose of *M*. *marinum*. AB fish were immunized intramuscularly with the experimental and control (GFP) antigens, followed by an intraperitoneal infection with ~40 cfu of *M*. *marinum*. Five weeks post-infection, the fish were euthanized, and their internal organs were dissected, homogenized and subjected to DNA extraction. The bacterial burden in each fish was determined by qPCR with *M*. *marinum* specific primers. The experimental cfu values in each experiment are normalized with the median cfu of the GFP controls of the same experiment. The lines represent median values, and the bars and whiskers the minimum and maximum values for each group, respectively. N = 10–29 per group. * p<0.05, ** p<0.01 (two-tailed Mann-Whitney test).

In most humans, a *M*. *tuberculosis* infection most often leads to a sub-clinical, latent infection, where the infection retains the potential to reactivate [68,69]. Ideally, a TB vaccine would prevent new infections; however, a more realistic goal would be a vaccine that helps the host to limit and control the infection and to prevent the dissemination into a fulminant disease [33]. In the adult zebrafish, a primary infection can be modelled by a low-dose *M*. *marinum* infection, which in most fish leads to a latent disease with stable bacterial counts [8]. To assess the efficacy of the selected antigens against a primary infection, the fish were first immunized with the experimental and control vaccine plasmids, and four weeks later i.p. infected with ~40 cfu of *M*. *marinum*. Five weeks post-infection, the fish were sacrificed and the bacterial burden of each fish was quantified by qPCR (Fig 5). To enable the comparison of data from multiple experiments without bias from variations in the basal levels, the bacterial count of each sample was normalized with the median cfu value of the GFP control group of the same experiment. The raw values of the bacterial counts in each sample compared to the control group(s) are shown in S1 Fig. While most of the 15 antigens tested did not affect the progression of the infection in terms of bacterial numbers and none of them was able to clear the infection completely, four of the candidate vaccines reduced the bacterial burden significantly (two-tailed Mann-Whitney test). These included RpfE, which led to an 88% reduction in median bacterial counts; together with two PE protein family members, PE5_1 and PE31, and the metabolic protein cdh, which reduced the bacterial burden by 56%, 50% and 62%, respectively.

### Vaccine efficiency against a high-dose *M*. *marinum* infection

**Fig 6:**
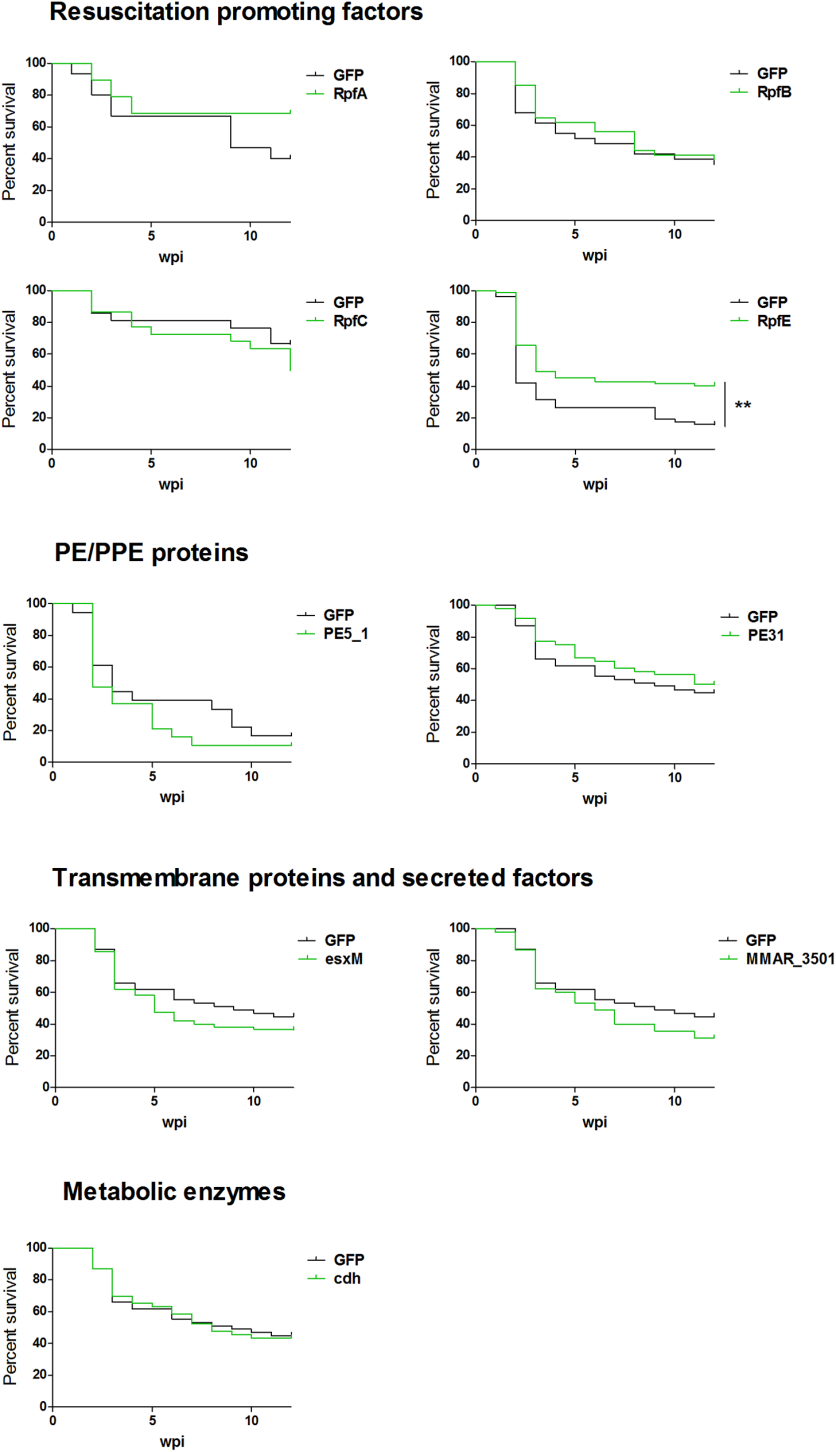
RpfE antigen improves survival of the fish infected with a high dose of *M*. *marinum*. AB fish were immunized intramuscularly with the experimental and control (GFP) antigens, followed by an intraperitoneal infection with ~10.000 cfu of *M*. *marinum*. Fish were then followed for 12 weeks for survival. The survival curve for each antigen immunization is shown separately with the GFP control group of the same infection experiment(s). ** p<0.01 (Log-rank (Mantel-Cox) test). N≥19 in each group.

In young children, a *M*. *tuberculosis* infection may lead to an acute, fulminant infection. The BCG vaccine protects children against this miliary TB, but due to safety issues, the use of BCG is limited in low-risk areas and excluded from HIV co-infected patients [1,5,35,69]. Therefore, a safer vaccine for preventing the dissemination of TB is required. To model a miliary TB infection in the adult zebrafish, we used a high-dose *M*. *marinum* infection that leads to an acute disease and relatively high mortality [8]. As a proof-of-concept, we have shown that zebrafish can be partially protected against a high-dose *M*. *marinum* infection by BCG vaccination, indicated by improved survival [38,39]. We used a similar approach to test the effect of the candidate DNA vaccines. Of the original 15 antigens, we chose 10 for assessment in a high-dose infection assay, including the four that significantly reduced the bacterial burden in the low-dose infection assay. As previously, the fish were immunized with the experimental and control antigens and infected with *M*. *marinum* five weeks later, this time with ~10.000 cfu. Survival of the fish was monitored for 12 weeks, during which all fish showing signs of disease were euthanized. The survival curves of each immunization compared with the control group of the same experiment are shown in Fig 6. One of the tested antigens, RpfE, led to a significantly improved survival (40% compared to the 16% of the control group). In addition, immunization with RpfA slightly enhanced fish survival from week 10 post infection onwards, although the effect was not statistically significant.

## Discussion

TB has a long history with mankind and it still remains a global challenge [70]. The bacterium has had time to evolve and adapt to its human host, and to develop means to avoid host immune responses or to use them for its own benefit [64,71]. Due to the complicated interactions between the bacterium and its host, proper *in vivo* models are needed for studying TB. The zebrafish, together with its natural pathogen *M*. *marinum*, have emerged as a feasible system to model TB [8–11]. Studies in zebrafish larvae and adults have shown several similarities in immune responses against mycobacterial infections in zebrafish and humans. These include the Toll-like receptor (TLR) signaling [18,19,72], leukotriene A_4_ hydrolase and the Tumor necrosis factor signaling [22,73,74], Th2 type cells [23,24] and lysosomal trafficking [25] and furin [26]. In addition, the zebrafish model has been used to study mycobacterial virulence factors and immune evasion strategies, revealing that many of them are used by both *M*. *marinum* and *M*. *tuberculosis*. Examples of this include the genes in the *RD1* locus [19,27,29]; the chemokine CXC-motive containing receptor 3 (CXCR3) signaling [20]; efflux pumps to achieve antibiotic tolerance [28], or the use of surface-associated membrane lipids to prevent the induction of TLR signaling [31]. Mycobacteria are also able to exploit the host’s resources for their own benefit, for example by inducing the expression of matrix metalloproteinase-9 (MMP9) in the host for the recruitment of macrophages [29] or by initiating granuloma-associated angiogenesis [30]. Consequently, the zebrafish model has already been used for designing novel drugs and therapies against TB [21,25,30,32]. Moreover, owing to its small size, fast production of offspring and relatively low housing costs, the zebrafish is also a suitable model for large scale biomedical screening studies [75]. Considering the scale of the global TB problem, the emergence of multi-drug resistant *M*. *tuberculosis* strains and the difficulty of predicting protective immune responses, the discovery of new drugs and vaccines likely will require such screening models [6].

Although attenuated, the BCG vaccine is a live pathogen, and thereby imposes a risk of a disseminated disease in immunocompromised individuals. This has limited its use in low-risk countries [35]. Tragically, the people in high-risk areas, who could benefit from the BCG vaccination, also have high a incidence of a co-infection with HIV, which prevents the use of BCG in these individuals [1,3]. Therefore, safer vaccine alternatives are being actively investigated and 14 candidates are currently in different phases of clinical trials. Subunit vaccines are generally considered safer than whole-cell vaccines, and several candidates are being studied at the moment [33]. The antigens chosen for a subunit vaccine depend on the intended protective category: a pre-exposure vaccine would contain antigens expressed in metabolically active and replicating *M*. *tuberculosis*, while a post-exposure vaccine would consist of antigens expressed during dormancy. As the subunit vaccine technology facilitates the use of several antigens, a combination of them would ideally give protection against both the active and latent stages of TB [33,76]. In our study, we tested 15 antigens that are expressed at different stages of the mycobacterium lifecycle and belong to different functional categories. We chose not to use BCG as a positive control because the most effective administration route for BCG in the zebrafish is an intraperitoneal injection, while DNA vaccines are injected intramuscularly. In addition, BCG is unable to replicate or form granulomas in the zebrafish and thus its protection is rather modest and variable [38, 39].

Prior to the screening in the infection assays, we verified the expression of the corresponding mycobacterial genes in the ATCC 927 strain by qRT-PCR. In the vaccine plasmid, the antigens were expressed as GFP fusion proteins, which facilitated the verification of their expression in vivo. For this, we used fluorescence microscopy, ELISA and Western blotting to allow the analysis of the expression of the antigens in situ, quantitatively and qualitatively, respectively. All of the fusion proteins were detected in each of the assays. As fluorescent microscopy allows the detection of antigen expression easily and without harming the fish, we used it to assess the success of each vaccination during the screening.

We used two assay settings to study the efficiency of the antigens against a mycobacterial infection: a low-dose infection followed by the quantification of the bacterial burden five weeks after infection, and a high-dose infection followed by the monitoring of survival for 12 weeks. The former is set to simulate a primary infection, and the latter a fulminant disease. As the stress caused to the fish by a high-dose infection and a survival assay is higher than that caused by a low-dose infection, for ethical reasons, we decided to exclude some of the antigens that did not show any protective effect against the low-dose infection from the survival study, even though the infection phases studied by the assays are different.

Four antigens were found to have protective effects against a low-dose mycobacterial infection. These include the probable CDP-diacylglycerol pyrophosphatase cdh, and two antigens belonging to the PE/PPE family, namely PE5_1 and PE31, and RpfE. Of these, cdh remains rather poorly characterized. Both the PE5_1 and PE31 antigens led to an approximately 50% reduction in the median cfu counts compared to the control group in the low-dose *M*. *marinum* infection assay. Their *M*. *tuberculosis* homologs, PE15 (Rv1386) and PE13 (Rv1195), have been studied using a recombinant *M*. *smegmatis* strain. Both recombinants led to the enhanced survival of bacteria within macrophages, presumably due to interference with host (innate) immune signaling pathways [77,78]. The expression of *pe13* was upregulated by diverse types of stress, and led to the increased production of interlukin-6 (IL-6) and IL-1β in macrophages [77], while PE15 upregulated anti-inflammatory cytokines and down-regulated proinflammatory cytokines and nitric oxide [78]. Thus, it is possible that both of these proteins are involved in evading the host immune response thereby promoting the survival of the mycobacteria. Further studies are required to determine the usefulness of these antigens as vaccine candidates. For example, they could be studied as a combination of two or more antigens, or if they are able to boost the protection offered by the BCG vaccination.

Of the mycobacterial antigens included in our screen, the Rpf proteins are probably the best studied, both considering their role in mycobacterial pathogenicity and their potential medical use. The latter is supported also by the results of this study, where RpfE was the only antigen that provided protection against both a primary (low dose) and a fulminant (high dose) infection. This is in line with previous results from mouse studies. In a mouse *ex vivo* model, RpfE induced the maturation of dendritic cells via the TLR4 leading to the generation of Th1 and Th17 cell mediated immunity, without stimulating the suppressive regulatory T cells [79]. RpfE has been also studied to some extent as a DNA vaccine candidate in the mouse model, where it has shown high immunogenicity and variable protection against *M*. *tuberculosis* both in terms of cfu burdens and survival times [76,80]. Considering that Rpf proteins are variably expressed during reactivation from dormancy, and that the *M*. *marinum* infection in adult zebrafish displays a natural latency that can be reactivated experimentally or spontaneously [8,23], the zebrafish model provides a promising platform to study Rpfs as vaccine candidates against the reactivation of latent TB. This is an important aspect in the TB research, as immunization of the latent *M*. *tuberculosis* carriers, especially adolescents and young adults, who are the main source of TB transmission, would effectively limit new infections [81]. We have previously shown that the adult zebrafish is partially protected against a *M*. *marinum* infection by the BCG vaccine [38,39], and that this protection can be boosted by immunization with a DNA vaccine consisting of RpfE combined with two other well-studied antigens ESAT-6 and Ag85 [39]. This makes the zebrafish a promising model for developing booster vaccines for BCG.

In conclusion, this study indicates that the *M*. *marinum* infection model in the adult zebrafish is suitable for early-stage pre-clinical TB vaccine screening and that the PE/PPE proteins and Resuscitation promoting factors, especially RpfE, are interesting candidates for further studies as antigens for DNA vaccines against TB.

## Supporting information

S1 Fig Bacterial counts in adult zebrafish after immunization and a low dose M. marinum infection. AB zebrafish were vaccinated intramuscularly with experimental antigens and a control (GFP), followed by an intraperitoneal *M*. *marinum* infection (~40 cfu). Five weeks post infections, fish were euthanized and their internal organs were collected for DNA extractions. Bacterial burdens were determined from the extracted DNAs by qPCR with *M*. *marinum* specific primers. Figures show the pooled results of different experiments, which are indicated with different colors. Each dot represents the bacterial count in one fish, and the horizontal lines represent median values. N = 10–29. * p<0.05 (two-tailed Mann-Whitney test). Abbreviations: PE5, PE5_1; PE_19, PE19_1; 4207, MMAR_4207; 3501, MMAR_3501.
(TIF)

S1 Table The primers used for qRT-PCR analysis. (DOCX)

S2 Table The primers used for cloning the antigens. (DOCX)
