# Supplementary figures and images for: Identification of novel antigen candidates for a tuberculosis vaccine in the adult zebrafish (Danio rerio)
Source: PLoS One. 2017 Jul 25;12(7):e0181942. doi: 10.1371/journal.pone.0181942 (PMC5526617; doi:10.1371/journal.pone.0181942)

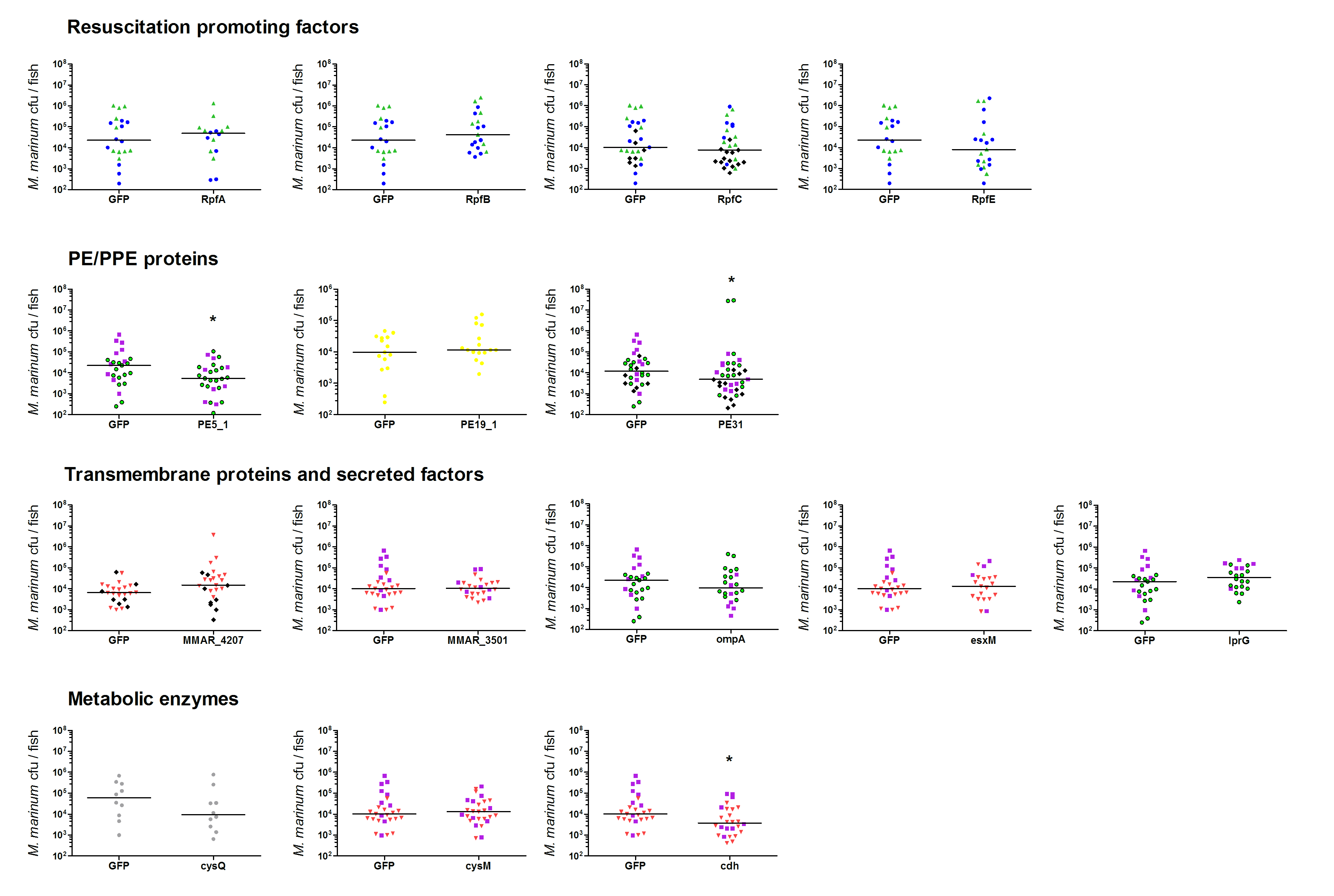

Supplement: S1 Fig — AB zebrafish were vaccinated intramuscularly with experimental antigens and a control (GFP), followed by an intraperitoneal M. marinum infection (~40 cfu). Five weeks post infections, fish were euthanized and their internal organs were collected for DNA extractions. Bacterial burdens were determined from the extracted DNAs by qPCR with M. marinum specific primers. Figures show the pooled results of different experiments, which are indicated with different colors. Each dot represents the bacterial count in one fish, and the horizontal lines represent median values. N = 10–29. * p<0.05 (two-tailed Mann-Whitney test). Abbreviations: PE5, PE5_1; PE_19, PE19_1; 4207, MMAR_4207; 3501, MMAR_3501. (TIF) [file pone.0181942.s001.tif]
