# Supplementary material for: Identification of novel antigen candidates for a tuberculosis vaccine in the adult zebrafish (Danio rerio)
Source: PLoS One. 2017 Jul 25;12(7):e0181942. doi: 10.1371/journal.pone.0181942 (PMC5526617; doi:10.1371/journal.pone.0181942)
Supplement: S1 Table — (DOCX) [file pone.0181942.s002.docx]

**S1 Table. The primers used for qRT-PCR analysis.**

| **Gene** | **Forward primer** | **Reverse primer** |
| --- | --- | --- |
| *RpfA* | ATGTCCACGAGGCTTCGACC | TCGTTGCCGCTGATGTTCTG |
| *RpfB* | AGTTTCTGCGTCCAAGACCG | CTGGACGATGTCGATCACCC |
| *RpfC* | CACCTGGGAGGAATACGGTG | TTGGCGATCGCGATTTGTTG |
| *RpfE* | CCAAGGTCTACACCGTGAACT | GTGTTGATGCCCCAGTTACC |
| *PE5_1* | GAGTTATTCCCGAGGGTTTG | GGATCACCGCCGTAATGG |
| *PE19_1* | AGATGTATCAAGCCGTGAGCG | GTACGACCCTGAGCTGATGC |
| *PE31* | GTCTATCGGAGCGGCATTGA | GCAAGCAGTTCGGAAACCTC |
| *MMAR_4207* | TTCGGCCTGATTACCTCAGC | ATCGCAGAGCACGTCATAGC |
| *MMAR_3501* | GTTGTGGTGCAAGTGCGATG | ATACTTGGATACGCTGGCCC |
| *ompA* | ATTTCTCCAACGCCGAACCT | AAGGTGATGGTGTCCCGTTC |
| *esxM* | CATGGGCCAGATGAACACCG | TGCTCTTGCTGCTCGTAGTTG |
| *lprG* | GATCACCGGAAAGGTCACTG | GTTCTCCTGAATCCACACGG |
| *cysQ* | CGGCAATCCTCTATCACGTC | AGATAGGCATCGGCTTTGC |
| *cysM* | TCTACGGGACCGAGATCATA | GGTAGAGCAGGACCCATTC |
| *cdh* | GGGTGGCCATAGTTATCACG | GCGTTTTCCATCTTCTTGCC |
| *MMITS* | CACCACGAGAAACACTCCAA | ACATCCCGAAACCAACAGAG |
