# Supplementary material for: Identification of novel antigen candidates for a tuberculosis vaccine in the adult zebrafish (Danio rerio)
Source: PLoS One. 2017 Jul 25;12(7):e0181942. doi: 10.1371/journal.pone.0181942 (PMC5526617; doi:10.1371/journal.pone.0181942)
Supplement: S2 Table — (DOCX) [file pone.0181942.s003.docx]

**S2 Table. The primers used for cloning the antigens.**

| **Gene** | **Forward primer** | **Reverse primer** |
| --- | --- | --- |
| *RpfA* | CACACAGCTAGCCACCATGGTCAGCGTCGCCAAGATC | CACACAGAATTCCGATCTTGGCGACGCTGAC |
| *RpfB* | CACACAGCTAGCCACCATGTTGCGCGCTGTGG | CAGCCAGAATTCCCACCGCGATCTGTTCTTC |
| *RpfC* | CACACAGCTAGCCACCATGACACACATCGCGAAAC | CACACAGAATTCGGGGCGTGAATATCGAGATG |
| *RpfE* | CACACAGCTAGCCACCATGAAGAACGTCCGCAACAG | CACACAGAATTCCTCGGATCTGCTGCTCTTCAC |
| *PE5_1* | ACACAGCTAGCCACCGTGATGTTGCGAGTTATTCCC | CACACAGAATTCCAAAAGCGCCGTAGCC |
| *PE19_1* | ACACAGCTAGCCACCATGCATACGTGACCACAC | ACACGAATTCCGTACGACCCTGAGCTG |
| *PE31* | ACATAGCTAGCCACCATGTCTTCTGTTACGGCTCGAC | TCAGAATTCCGGTTTCGGTGCTCAGG |
| *MMAR_4207* | ACATAGCTAGCCACCATGACTAGCCCCTTCCAG | ACGAATTCGATCGCAGAGCACGTCATAGC |
| *MMAR_3501* | ATATAGCTAGCCACCATGGAGACGTTCAAACTGGAC | ACGAATTCCGTCTTGCAGACAACTGAGC |
| *ompA* | CACACAGCTAGCCACCATGGTGGGTACCGACGCGG | CACACAGAATTCCCACAATTTCGACACGGC |
| *esxM* | ACATAGCTAGCCACCATGACTGCACGCTTTATGAC | ACGAATTCCCTGCTCTTGCTGCTCG |
| *lprG* | ATATAGCTAGCCACCATGAAGACGCTCTCCGGC | ACGAATTCTGGACAAGGTCATCTCGACG |
| *cysQ* | ATATAGCTAGCCACCATGCGGGAAGAGGTTGG | ACGAATTCCATCGAGCAGCACAGGC |
| *cysM* | ATATAGCTAGCCACCATGACCCGCTACGACTCAC | ACGAATTCTAGGTGCCGGGTGTTCATA |
| *cdh* | CACACAGCTAGCCCACCATGGCCGACCGGGATCAAC | CACACAGAATTCCTTTGGCGACCGAGCAG |
